# Supplementary material for: Disengagement from treatment and its socio-demographic and clinical predictors among patients with incident schizophrenia in a Nigerian psychiatric hospital: 8-year naturalistic follow-up analysis
Source: PLOS Ment Health. 2025 Mar 12;2(3):e0000251. doi: 10.1371/journal.pmen.0000251 (PMC12798387; doi:10.1371/journal.pmen.0000251)
Supplement: Attachment — Submitted filename: Response to the in-house editorial comments.docx [file pmen.0000251.s003.docx]

Thank you for submitting your manuscript entitled "Disengagement from Treatment and its Socio-demographic and Clinical Predictors among Patients with Incident Schizophrenia in a Nigerian Psychiatric Hospital: 8-Year Naturalistic Follow-up Analysis" to PLOS Mental Health. Your manuscript files have been checked in-house, but before we can proceed we need you to address the following issues:

Thank you for clarifying how the capacity to provide consent was determined .

We also kindly request you to provide explanation if your ethics committee or IRB approved this consent procedure.

Kindly do not remove the uploaded files. Thank you for your attention. We look forward to hearing from you.

**Authors’ Response:** This to confirm that the Ethics and Research Committee our institution approved this consent procedure.
